# Perioperative CALLY dynamics after curative gastrectomy for gastric adenocarcinoma: associations with nodal tumor burden, textbook surgical outcome, and disease-free survival

**DOI:** 10.3389/fonc.2026.1915467

**Published:** 2026-09-03

**Authors:** Adem Ozcan, Gizem Gunes

**Affiliations:** Department of Surgical Oncology, Ankara Bilkent City Hospital, Ankara, Türkiye

**Keywords:** CALLY index, disease-free survival, gastrectomy, gastric adenocarcinoma, LODDS, lymph node ratio, nodal tumor burden, textbook surgical outcome

## Abstract

**Background:**

The C-reactive protein–albumin–lymphocyte index (CALLY) integrates inflammation, nutrition, and cellular immunity. Its relationships with nodal tumor burden and within-patient postoperative recovery after gastrectomy remain unclear.

**Methods:**

This retrospective cohort included 188 patients undergoing R0 gastrectomy for non-metastatic gastric adenocarcinoma. Preoperative CALLY was evaluated in relation to metastatic lymph node count, lymph node ratio (LNR), log odds of positive lymph nodes (LODDS), and pN status. The relationships of perioperative CALLY with major postoperative complications and textbook surgical outcome were evaluated using longitudinal CALLY recovery ratios in 178 patients who underwent open gastrectomy. Primary disease-free survival (DFS) and overall survival (OS) analyses included 165 patients operated on or before 31 May 2025.

**Results:**

The median preoperative CALLY was 0.572. Low CALLY was independently associated with high LNR (adjusted odds ratio [aOR], 2.03; 95% CI, 1.04–3.97; p = 0.037) and high LODDS (aOR, 2.11; 95% CI, 1.08–4.14; p = 0.029). Major complications occurred in 40 patients (21.3%), and textbook surgical outcome was achieved in 139 (73.9%). In the open-gastrectomy cohort, CALLY trajectories differed by major complication and textbook surgical outcome status (both interaction p < 0.001). At postoperative day 7 (POD7), recovery ratios were 0.0115 versus 0.0932 in patients with versus without major complications and 0.0156 versus 0.1019 in patients who failed versus achieved textbook surgical outcome (both p < 0.001). During a mean observed follow-up of 27.4 ± 10.6 months, 52 DFS events occurred. Low CALLY was associated with worse DFS (log-rank p = 0.029; univariable hazard ratio, 1.85; 95% CI, 1.06–3.23), whereas the continuous association was attenuated after multivariable adjustment (adjusted hazard ratio, 0.58; 95% CI, 0.33–1.04; p = 0.067). OS did not differ significantly (log-rank p = 0.126).

**Conclusion:**

Preoperative CALLY was associated with nodal tumor burden, whereas impaired postoperative CALLY recovery reflected adverse postoperative courses. Postoperative CALLY should be interpreted as a monitoring index rather than a predictive biomarker. The DFS association requires external validation.

## Introduction

Gastric cancer remains a major cause of cancer-related mortality worldwide despite advances in perioperative systemic therapy, surgical technique, pathological staging, and postoperative care (1–4). For patients with resectable gastric adenocarcinoma, curative-intent gastrectomy with adequate lymphadenectomy remains the cornerstone of treatment. Nevertheless, outcomes after curative resection remain heterogeneous. Patients with comparable anatomical stage may differ substantially in nodal tumor burden, postoperative recovery, recurrence risk, and survival, indicating that conventional staging alone does not fully capture the interaction between tumor biology and host-related factors.

Nodal tumor burden is one of the most important determinants of prognosis in gastric adenocarcinoma. Although the AJCC pN category is based on the absolute number of metastatic lymph nodes, its prognostic accuracy may be influenced by the total number of retrieved lymph nodes and the quality of surgical-pathological assessment. Complementary nodal metrics such as lymph node ratio (LNR) and log odds of positive lymph nodes (LODDS) have therefore been proposed to refine risk stratification after gastrectomy (5–7). These metrics provide a more quantitative representation of lymphatic dissemination and may better reflect the biological aggressiveness of the disease.

The host systemic inflammatory, nutritional, and immune status has also emerged as a clinically relevant determinant of oncological and perioperative outcomes. Cancer-related inflammation can promote tumor progression, immune escape, and metastatic potential, whereas malnutrition and impaired cellular immunity may reduce tolerance to major surgery and systemic therapy. Several routinely available blood-based scores, including the Prognostic Nutritional Index (PNI), neutrophil-to-lymphocyte ratio (NLR), platelet-to-lymphocyte ratio (PLR), and modified Glasgow Prognostic Score (mGPS), have been evaluated in gastric cancer. However, these indices usually reflect only selected components of the host response.

The C-reactive protein–albumin–lymphocyte index (CALLY) integrates three biologically relevant domains: systemic inflammation, nutritional reserve, and cellular immune competence. Recent studies and meta-analyses suggest that low CALLY is associated with adverse clinicopathological features, postoperative morbidity, recurrence, and poorer survival in gastric cancer (8–13). However, most available evidence has focused on CALLY as a static preoperative prognostic marker. Its relationship with quantitative nodal tumor burden, particularly LNR and LODDS, and its association with composite surgical quality outcomes after curative gastrectomy remain incompletely defined.

The early postoperative period may provide additional biological information beyond baseline risk assessment. Gastrectomy induces a measurable inflammatory and catabolic response characterized by CRP elevation, albumin decline, and lymphocyte suppression. These changes may reflect the magnitude of surgical stress, occult postoperative complications, nutritional depletion, and the patient’s capacity for immune-metabolic recovery. Postoperative inflammatory-nutritional markers, including CRP-based indices and NLR, have been associated with postoperative morbidity and long-term outcomes after gastric cancer surgery (14, 15). Therefore, serial perioperative CALLY assessment may offer a dynamic view of postoperative inflammatory-nutritional-immune recovery after gastrectomy.

Textbook outcome has gained increasing attention as a multidimensional measure of ideal surgical care in gastric cancer. Unlike isolated endpoints, it combines technical, pathological, and recovery-related outcomes such as adequate lymph node retrieval, absence of major complications, no anastomotic leakage, no early mortality, no reoperation or readmission, and acceptable postoperative length of stay (16, 17). A perioperative marker that captures preoperative vulnerability or an adverse postoperative recovery trajectory could support individualized risk assessment and clinical monitoring.

In this study, we evaluated the clinical significance of perioperative CALLY in patients undergoing curative-intent gastrectomy for gastric adenocarcinoma. The primary objective was to investigate the relationship between preoperative CALLY and quantitative nodal tumor burden, including metastatic lymph node count, LNR, LODDS, pN positivity, and N3 disease. Secondary objectives were to characterize within-patient postoperative CALLY trajectories and examine their associations with major postoperative complications and textbook surgical outcome. DFS and OS were evaluated in a follow-up-mature cohort with at least 12 months of potential observation. We hypothesized that low preoperative CALLY would be associated with greater nodal tumor burden, whereas impaired postoperative CALLY recovery would be associated with an adverse postoperative course.

## Materials and methods

### Study design and patients

This retrospective, single-center observational cohort study included consecutive adult patients who underwent R0 curative-intent gastrectomy for histopathologically confirmed non-metastatic gastric adenocarcinoma at Ankara Bilkent City Hospital between 12 October 2020 and 14 April 2026. Patients undergoing emergency surgery, palliative or other non-curative resection, or surgery for metastatic disease, as well as patients with synchronous malignancy or missing essential laboratory, pathological, postoperative, or follow-up data, were excluded. The study was conducted in accordance with the Declaration of Helsinki and was approved by the Ankara Bilkent City Hospital Medical Research Scientific and Ethical Evaluation Committee (TABED 1-26-2580; 20 May 2026). The requirement for study-specific informed consent was waived because of the retrospective design and use of anonymized routine-care data. The study was reported in accordance with the STROBE recommendations.

The database was locked on 31 May 2026. All eligible patients were included in clinicopathological, nodal, and perioperative analyses. For the primary time-to-event analyses, a follow-up-mature cohort was defined by including patients who underwent curative gastrectomy on or before 31 May 2025. This calendar-based definition ensured at least 12 months of potential follow-up for event-free patients and was independent of CALLY status and subsequent outcomes.

### Data collection and biomarker assessment

Demographic, clinical, operative, laboratory, pathological, and follow-up data were retrieved from the institutional electronic medical records, operative reports, pathology reports, laboratory databases, and outpatient records. Variables included age, sex, American Society of Anesthesiologists classification, Eastern Cooperative Oncology Group performance status, neoadjuvant treatment, type and approach of gastrectomy, tumor size, histological type, grade, Lauren classification, lymphovascular and perineural invasion, pathological T and N categories, AJCC stage group, retrieved and metastatic lymph node counts, postoperative complications, length of stay, readmission, reoperation, recurrence, and survival status. Pathological stage was recorded as pTNM in patients undergoing upfront surgery and as ypTNM in patients receiving neoadjuvant treatment, according to the AJCC eighth edition.

CALLY was calculated as albumin × lymphocyte count/CRP, with albumin expressed in g/dL, CRP in mg/L, and lymphocyte count in 10^9^/L. NLR, PLR, PNI, and mGPS were calculated using established definitions. CALLY was calculated preoperatively, on postoperative days 1, 3, and 7 (POD1, POD3, and POD7), and at discharge. For patients discharged before POD7, the POD7 sample was obtained during the scheduled outpatient assessment around postoperative day 7. No laboratory measurement was performed solely for research purposes.

To quantify within-patient postoperative change, a CALLY recovery ratio was calculated at each postoperative time point by dividing the postoperative CALLY value by the corresponding preoperative value. Values below 1 indicated suppression relative to baseline.

### Outcomes and follow-up

The primary endpoint was nodal tumor burden, evaluated using metastatic lymph node count, pN positivity, N3 disease, LNR, and LODDS. LNR was calculated as the number of metastatic lymph nodes divided by the total number of retrieved lymph nodes. LODDS was calculated as ln[(metastatic lymph nodes + 0.5)/(negative lymph nodes + 0.5)].

Postoperative complications occurring within 30 days were graded according to the Clavien–Dindo classification, and grade III or higher complications were considered major (18, 19). Textbook surgical outcome was defined using an adapted gastrectomy-specific composite comprising R0 resection, retrieval of at least 15 lymph nodes, absence of major complications and anastomotic leakage, no 30-day reoperation or readmission, no 30- or 90-day mortality, and postoperative length of stay <21 days (16). Because R0 resection was an eligibility criterion, the R0 component was uniformly fulfilled by all patients in the analytic cohort. Analyses using the original, more restrictive length-of-stay threshold of ≤11 days were retained as sensitivity analyses (**Supplementary Table 7**).

Recurrence was defined as the first radiologically, pathologically, endoscopically, surgically, or multidisciplinary-documented locoregional, peritoneal, or distant recurrence. DFS was measured from curative gastrectomy to recurrence, death from any cause, or last follow-up. OS was measured from curative gastrectomy to death from any cause or last follow-up. Accordingly, a DFS event comprised either documented recurrence or death without previously documented recurrence.

### Statistical analysis

Statistical analyses were performed using IBM SPSS Statistics for Windows, version 31.0 (IBM Corp., Armonk, NY, USA), and Python version 3.13.5 with SciPy 1.17.0, statsmodels 0.14.6, and scikit-learn 1.8.0.

Continuous variables were summarized as mean ± standard deviation or median with interquartile range, according to distribution, and categorical variables as number and percentage. Between-group comparisons were performed using Student’s t test, Mann–Whitney U test, Pearson chi-square test, or Fisher’s exact test, as appropriate.

Preoperative CALLY was analyzed continuously and after dichotomization using the full-cohort median of 0.572. This full-cohort median threshold was retained in all subgroup and sensitivity analyses. Relationships with metastatic lymph node count, LNR, and LODDS were evaluated using Spearman correlation. Logistic regression models were constructed for pN-positive disease, high LNR, and high LODDS. High LNR and high LODDS were defined as values above their exact full-cohort medians; the corresponding thresholds are displayed as 0.0526 and −2.605, respectively, for presentation. Multivariable models included age, sex, neoadjuvant treatment, advanced T stage, lymphovascular invasion, and perineural invasion. Overlapping nodal and stage variables were not entered simultaneously.

Because surgical approach may influence the postoperative inflammatory response, primary longitudinal CALLY analyses were restricted to patients undergoing open gastrectomy. Postoperative recovery ratios were natural-log transformed and analyzed using generalized estimating equations with patient identification as the clustering variable, an exchangeable working correlation structure, perioperative time as a categorical within-patient factor, outcome group as a between-patient factor, and a time-by-outcome interaction term. Separate models were constructed for major complications and textbook surgical outcome failure. Corresponding analyses in the complete cohort were performed as sensitivity analyses. The preoperative reference value of 1.0 was used solely for normalization and graphical presentation and was not included as a repeated time point in the GEE models. Accordingly, the longitudinal GEE analyses included POD1, POD3, POD7, and discharge, yielding three degrees of freedom for the time-by-outcome interaction.

Primary DFS and OS analyses were performed in the follow-up-mature cohort. Survival curves were estimated using the Kaplan–Meier method and compared using the log-rank test. Observed follow-up was calculated from the date of curative gastrectomy to death or last documented follow-up and was summarized as mean ± standard deviation. Numbers at risk were displayed at 12-month intervals below each Kaplan–Meier curve. Cox proportional hazards models were used to estimate hazard ratios with 95% confidence intervals. The multivariable DFS model included continuous preoperative CALLY, stage III disease, lymphovascular invasion, perineural invasion, and major postoperative complications. The proportional hazards assumption was assessed using Schoenfeld residuals and log-minus-log survival plots. Complete-cohort survival analyses and analyses restricted to patients with an observed postoperative follow-up of at least 12 months were performed as sensitivity analyses. Exploratory receiver operating characteristic analyses were performed for selected binary endpoints and are reported in the **Supplementary Material**. Optimal thresholds were identified using the Youden index, and Areas under the receiver operating characteristic curves (AUCs) were reported with 95% confidence intervals estimated by bootstrap resampling. To compare the discriminatory performance of CALLY with its individual components, ROC AUCs for CALLY were compared with those for CRP, albumin, and lymphocyte count for selected preoperative and postoperative endpoints. Marker directions were oriented so that higher scores indicated higher-risk status: CRP was analyzed directly, whereas inverse CALLY, albumin, and lymphocyte values were used. Pairwise AUC differences between CALLY and each component were evaluated using 5,000 stratified bootstrap resamples. Because CALLY is algebraically derived from CRP, albumin, and lymphocyte count, the composite index and its individual components were not entered simultaneously in the same multivariable model. Complete-case analyses were performed, and no data were imputed. No adjustment for multiple comparisons was applied to secondary or exploratory analyses; these findings were therefore interpreted as hypothesis-generating. A two-sided p value <0.05 was considered statistically significant.

## Results

### Cohort characteristics

**Figure 1:**
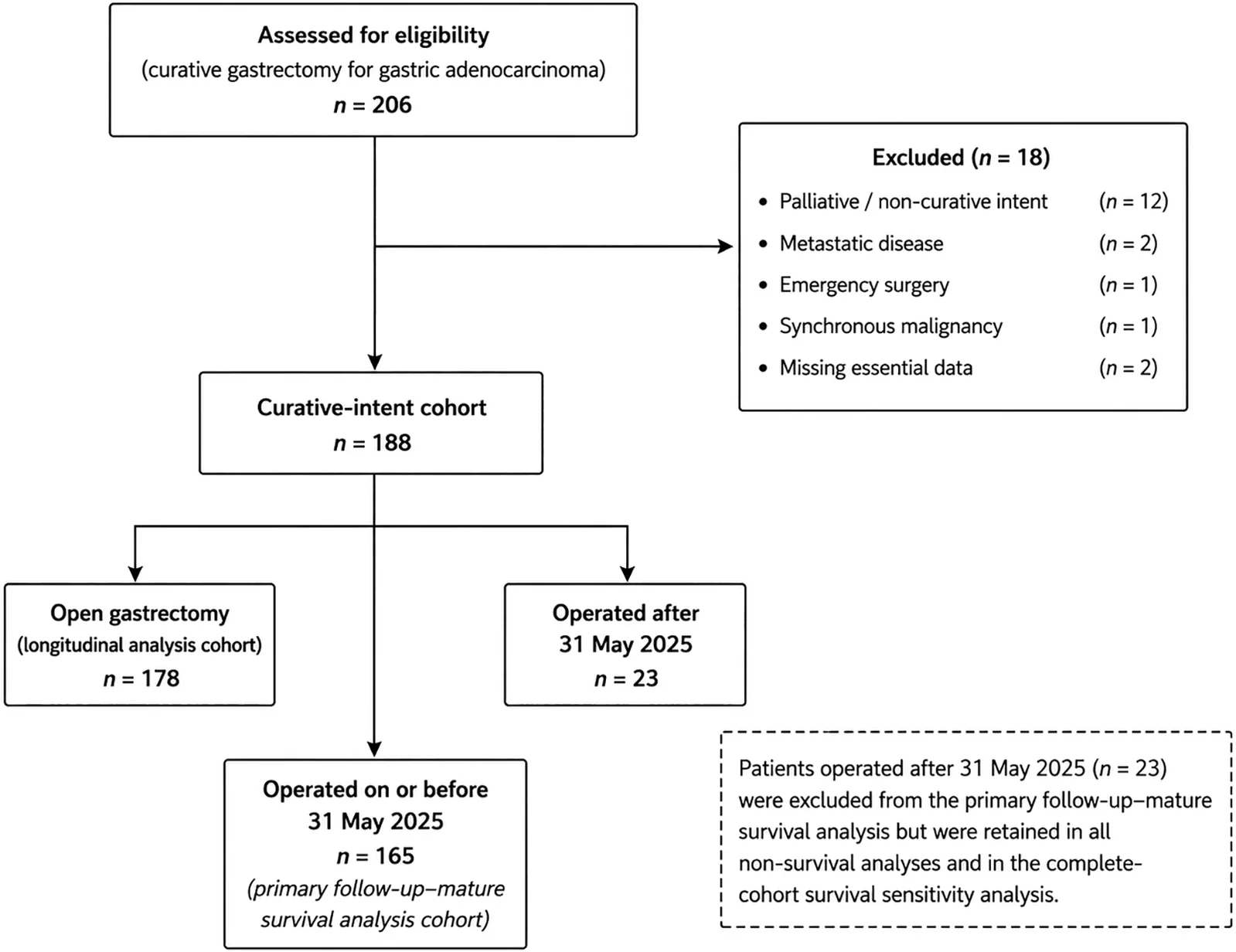
Patient selection and analytic cohorts. A total of 206 patients undergoing gastrectomy for gastric adenocarcinoma during the study period were assessed for eligibility. Eighteen patients were excluded: 12 underwent palliative or non-curative resection, 2 had metastatic disease at surgery, 1 underwent emergency surgery, 1 had a synchronous malignancy, and 2 had missing essential data. The final curative-intent cohort comprised 188 patients and was used for clinicopathological, nodal, and perioperative analyses. Of these, 178 patients who underwent open gastrectomy constituted the primary longitudinal CALLY cohort. One hundred sixty-five patients who underwent surgery on or before 31 May 2025 constituted the follow-up-mature cohort for the primary survival analyses. The 23 patients operated after 31 May 2025 were excluded from the primary survival analysis but retained in all non-survival analyses and in the complete-cohort survival sensitivity analysis. CALLY, C-reactive protein–albumin–lymphocyte index; DFS, disease-free survival; OS, overall survival.

A total of 206 patients were assessed for eligibility. Eighteen patients were excluded because of palliative or non-curative resection (n=12), metastatic disease at surgery (n=2), emergency surgery (n=1), synchronous malignancy (n=1), or missing essential data (n=2). The remaining 188 patients constituted the complete curative-intent cohort and were included in clinicopathological, nodal, and perioperative analyses (**Figure 1**). The median age was 67 years, and 114 patients (60.6%) were male. Major postoperative complications occurred in 40 patients (21.3%). Using the adapted definition, textbook surgical outcome was achieved in 139 patients (73.9%).

The median preoperative CALLY was 0.572, resulting in 94 patients in each low- and high-CALLY group. Age, sex, ASA classification, ECOG performance status, neoadjuvant treatment, and surgical approach were comparable between groups. In contrast, patients with low CALLY had larger tumors than those with high CALLY (median, 56.0 vs 42.5 mm), more frequent stage III disease (57.4% vs 25.5%), and more frequent serosal invasion (40.4% vs 3.2%; all p < 0.001). Selected baseline characteristics are presented in **Table 1**, while complete clinical, laboratory, and pathological characteristics are provided in **Supplementary Tables S1** and **S2**.

**Table 1:** Selected baseline clinical and pathological characteristics according to preoperative CALLY group.

Variable	Overall (n=188)	Low CALLY (n=94)	High CALLY (n=94)	p value
Age, years	67.0 (57.0–74.3)	67.5 (55.3–75.0)	67.0 (57.3–73.8)	0.874
Male sex	114 (60.6)	59 (62.8)	55 (58.5)	0.550
ASA III–IV	99 (52.7)	51 (54.3)	48 (51.1)	0.661
ECOG performance status ≥1	143 (76.1)	74 (78.7)	69 (73.4)	0.393
Neoadjuvant chemotherapy	36 (19.1)	17 (18.1)	19 (20.2)	0.711
Type of gastrectomy				0.050
Subtotal distal	90 (47.9)	49 (52.1)	41 (43.6)	
Total	86 (45.7)	36 (38.3)	50 (53.2)	
Proximal	12 (6.4)	9 (9.6)	3 (3.2)	
Open surgical approach	178 (94.7)	91 (96.8)	87 (92.6)	0.194
Tumor size, mm	49.5 (33.0–63.3)	56.0 (42.3–74.5)	42.5 (30.0–53.0)	<0.001
Stage III disease	78 (41.5)	54 (57.4)	24 (25.5)	<0.001
Lymphovascular invasion	68 (36.2)	40 (42.6)	28 (29.8)	0.095
Perineural invasion	77 (41.0)	44 (46.8)	33 (35.1)	0.138
Serosal invasion	41 (21.8)	38 (40.4)	3 (3.2)	<0.001

Data are presented as median (interquartile range) or n (%). Low and high CALLY groups were defined using the full-cohort median of 0.572. ASA, American Society of Anesthesiologists; ECOG, Eastern Cooperative Oncology Group.

One hundred sixty-five patients underwent surgery on or before 31 May 2025 and constituted the primary follow-up-mature survival cohort. Twenty-three patients operated after this date were excluded from the primary survival analysis but remained included in all non-survival analyses and in the complete-cohort survival sensitivity analysis.

### Preoperative CALLY and nodal tumor burden

**Figure 2:**
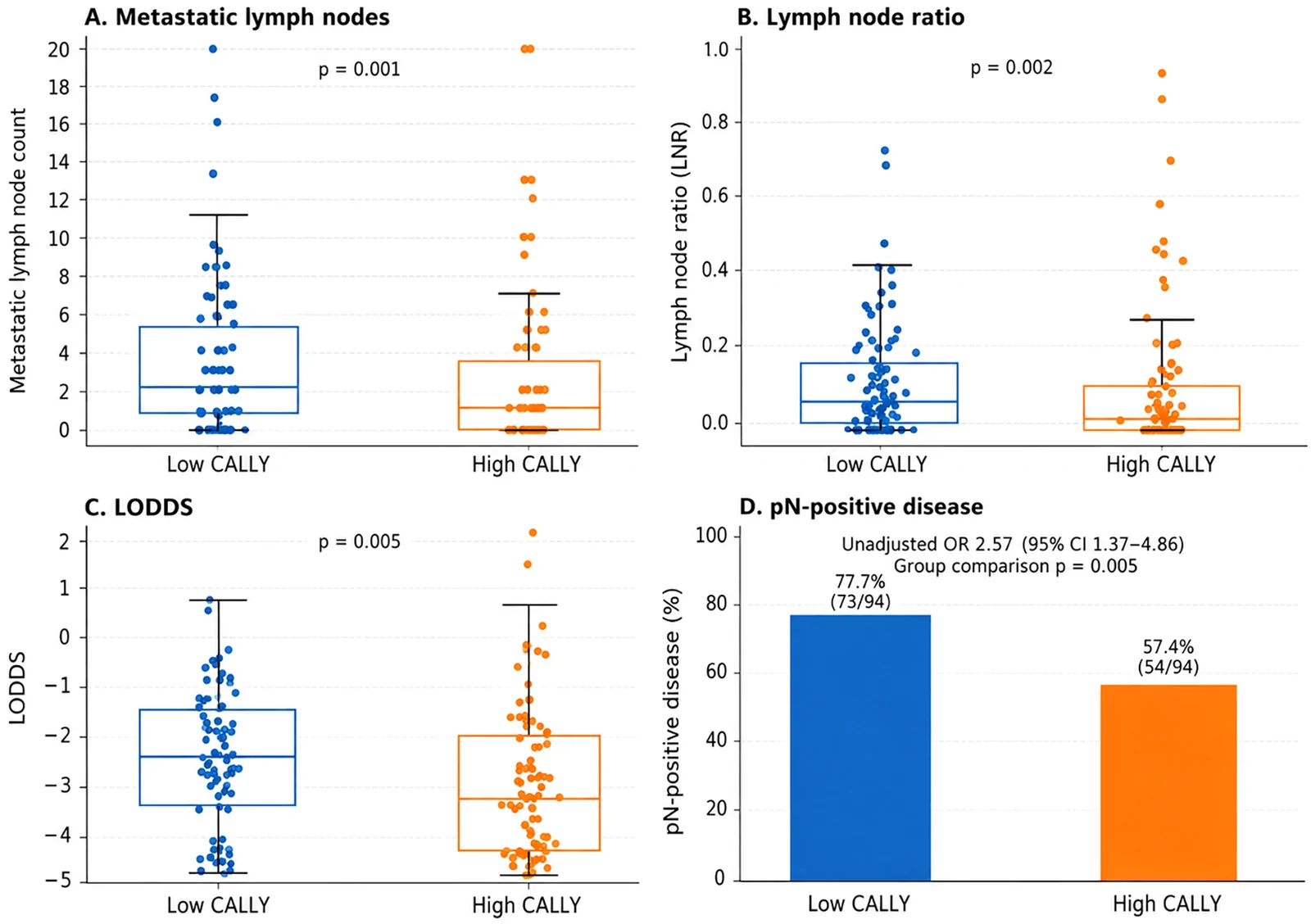
Association between preoperative CALLY and nodal tumor burden. Patients with low preoperative CALLY had higher metastatic lymph node counts **(A)**, higher lymph node ratio values **(B)**, higher LODDS values **(C)**, and more frequent pN-positive disease **(D)** than patients with high preoperative CALLY. Boxes represent interquartile ranges, horizontal lines represent medians, and individual points represent patients. The odds ratio shown in panel D is unadjusted; adjusted estimates are presented in **Table 2**. CALLY, C-reactive protein–albumin–lymphocyte index; LNR, lymph node ratio; LODDS, log odds of positive lymph nodes; OR, odds ratio; CI, confidence interval.

Low preoperative CALLY was associated with greater nodal tumor burden. Compared with the high-CALLY group, patients with low CALLY had higher metastatic lymph node counts, LNR, and LODDS and more frequent pN-positive disease, whereas the number of retrieved lymph nodes and the frequency of N3 disease did not differ significantly (**Table 2**, **Figure 2**).

**Table 2:** Preoperative CALLY and nodal tumor burden.

A. Group comparisons				
Nodal endpoint	Overall (n=188)	Low CALLY (n=94)	High CALLY (n=94)	p value
Retrieved lymph nodes	31 (26–35)	31 (26–35)	31 (26–35)	0.624
Metastatic lymph nodes	2 (0–4)	2 (1–5)	1 (0–3.5)	0.001
pN-positive disease	127 (67.6)	73 (77.7)	54 (57.4)	0.005
N3 disease	23 (12.2)	13 (13.8)	10 (10.6)	0.656
Lymph node ratio	0.053 (0–0.154)	0.077 (0.027–0.194)	0.029 (0–0.119)	0.002
LODDS	−2.605 (−3.980 to −1.609)	−2.282 (−3.205 to −1.371)	−3.106 (−4.111 to −1.897)	0.005
B. Adjusted associations between low preoperative CALLY and nodal endpoints				
Dependent variable	Events	Adjusted odds ratio (95% CI)		p value
pN-positive disease	127/188	1.62 (0.77–3.39)		0.200
High LNR	93/188	2.03 (1.04–3.97)		0.037
High LODDS	94/188	2.11 (1.08–4.14)		0.029

High LNR and high LODDS were defined as values above their exact full-cohort medians, displayed as 0.0526 and −2.605, respectively. Models were adjusted for age, sex, neoadjuvant treatment, advanced T stage, lymphovascular invasion, and perineural invasion. LNR, lymph node ratio; LODDS, log odds of positive lymph nodes; CI, confidence interval.

Preoperative CALLY correlated inversely with metastatic lymph node count (Spearman ρ=−0.206; p=0.005), LNR (ρ=−0.197; p=0.007), and LODDS (ρ=−0.181; p=0.013). After adjustment for age, sex, neoadjuvant treatment, advanced T stage, lymphovascular invasion, and perineural invasion, low CALLY remained associated with high LNR (aOR, 2.03; 95% CI, 1.04–3.97; p=0.037) and high LODDS (aOR, 2.11; 95% CI, 1.08–4.14; p=0.029), but not independently with pN-positive disease (aOR, 1.62; 95% CI, 0.77–3.39; p=0.200). Detailed covariate-level regression results are provided in **Supplementary Table 5**.

### Longitudinal perioperative CALLY dynamics

**Figure 3:**
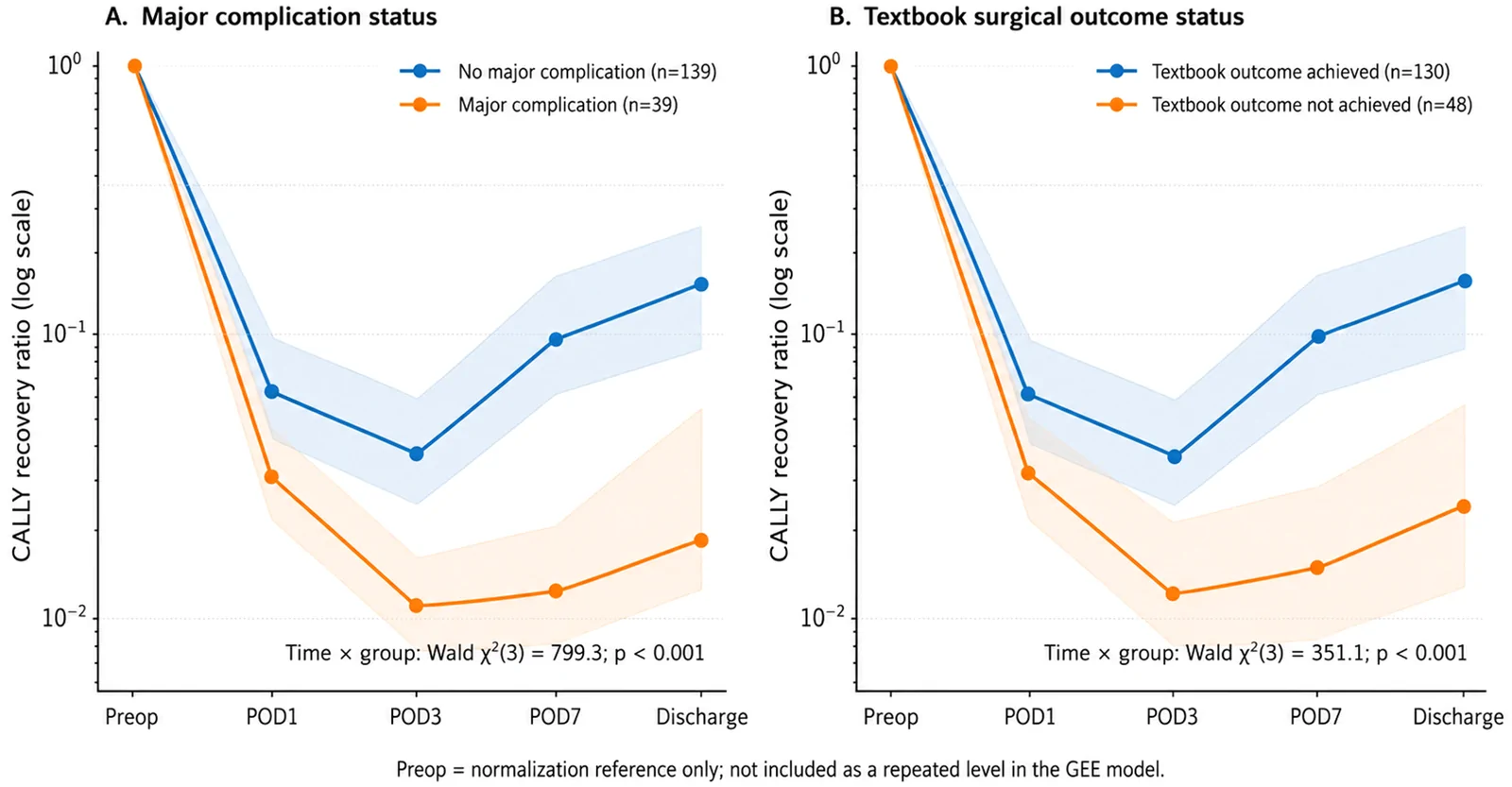
Within-patient perioperative CALLY recovery after open gastrectomy. Median CALLY recovery ratios with interquartile ranges are shown at POD1, POD3, POD7, and discharge according to major postoperative complication status **(A)** and textbook surgical outcome status **(B)**. Recovery ratios were calculated as postoperative CALLY divided by the corresponding preoperative CALLY value. The preoperative value of 1.0 is shown solely as the normalization reference and was not included as a repeated level in the GEE models. Shaded bands represent interquartile ranges. Perioperative trajectories differed significantly according to major complication and textbook surgical outcome status (both time-by-group interaction p<0.001). CALLY, C-reactive protein–albumin–lymphocyte index; POD, postoperative day; GEE, generalized estimating equations.

Primary longitudinal analyses included 178 patients who underwent open gastrectomy. Preoperative CALLY did not differ significantly according to major complication status or adapted textbook surgical outcome status. In contrast, within-patient postoperative CALLY trajectories differed significantly according to both major complication status and textbook surgical outcome status (both time-by-group interaction p<0.001; **Table 3**, **Figure 3**). Absolute perioperative CALLY values are provided in **Supplementary Table 3**.

**Table 3:** Longitudinal CALLY recovery after open gastrectomy.

A. Major postoperative complication status			
Time point	No major complication (n=139)	Major complication (n=39)
POD1/preoperative CALLY	0.0605 (0.0411–0.0936)	0.0308 (0.0222–0.0467)
POD3/preoperative CALLY	0.0372 (0.0254–0.0574)	0.0104 (0.0073–0.0158)
POD7/preoperative CALLY	0.0932 (0.0591–0.1505)	0.0115 (0.0074–0.0186)
Discharge/preoperative CALLY	0.1501 (0.0875–0.2301)	0.0176 (0.0119–0.0494)
B. Textbook surgical outcome status			
Time point	Achieved (n=130)	Not achieved (n=48)
POD1/preoperative CALLY	0.0611 (0.0410–0.0945)	0.0332 (0.0236–0.0519)
POD3/preoperative CALLY	0.0377 (0.0259–0.0610)	0.0125 (0.0076–0.0224)
POD7/preoperative CALLY	0.1019 (0.0620–0.1625)	0.0156 (0.0082–0.0312)
Discharge/preoperative CALLY	0.1606 (0.0920–0.2389)	0.0264 (0.0135–0.0600)

Time-by-group interaction: Wald χ²(3)=799.3; p<0.001.

Time-by-group interaction: Wald χ²(3)=351.1; p<0.001.

Data are median (interquartile range). CALLY recovery ratio was calculated as postoperative CALLY divided by preoperative CALLY. Analyses were restricted to the 178 patients undergoing open gastrectomy. POD, postoperative day. Primary inference was based on the longitudinal time-by-group interaction; individual time-point summaries are descriptive.

At POD7, the median recovery ratio was 0.0115 (IQR, 0.0074–0.0186) in patients with major complications and 0.0932 (IQR, 0.0591–0.1505) in those without major complications (p<0.001). The corresponding ratios were 0.0156 (IQR, 0.0082–0.0312) among patients who failed to achieve textbook surgical outcome and 0.1019 (IQR, 0.0620–0.1625) among those who achieved it (p<0.001).

Thirty-four of the 39 major complications in the open-gastrectomy cohort were documented by POD7. Accordingly, POD7 and discharge CALLY were interpreted as concurrent monitoring markers of postoperative recovery rather than prospective predictive biomarkers. Results were directionally unchanged in the complete-cohort sensitivity analysis (**Supplementary Table 6**). Exploratory ROC analyses are presented in **Supplementary Table 4** and **Supplementary Figure 1**. Paired comparisons between CALLY and its individual components are presented in **Supplementary Table 9**. For preoperative endpoints, CALLY showed numerically higher AUCs than CRP, albumin, or lymphocyte count; however, its differences from CRP and albumin were not statistically significant. CALLY showed higher discrimination than lymphocyte count for pN-positive disease, high LNR, and high LODDS. In the open-gastrectomy cohort, CRP showed higher discrimination than CALLY for major complications at POD1 and POD3, whereas no significant difference was observed at POD7 and CALLY showed higher discrimination at discharge. For adapted textbook surgical outcome failure, CRP showed higher discrimination at POD1, no significant differences were observed at POD3 or POD7, and CALLY showed higher discrimination at discharge.

### Survival outcomes

In the follow-up-mature cohort, the mean observed follow-up was 834.8 ± 322.2 days (27.4 ± 10.6 months). Forty-one patients developed documented recurrence, and 24 died. The 52 DFS events comprised 41 documented recurrences and 11 deaths without previously documented recurrence.

**Figure 4:**
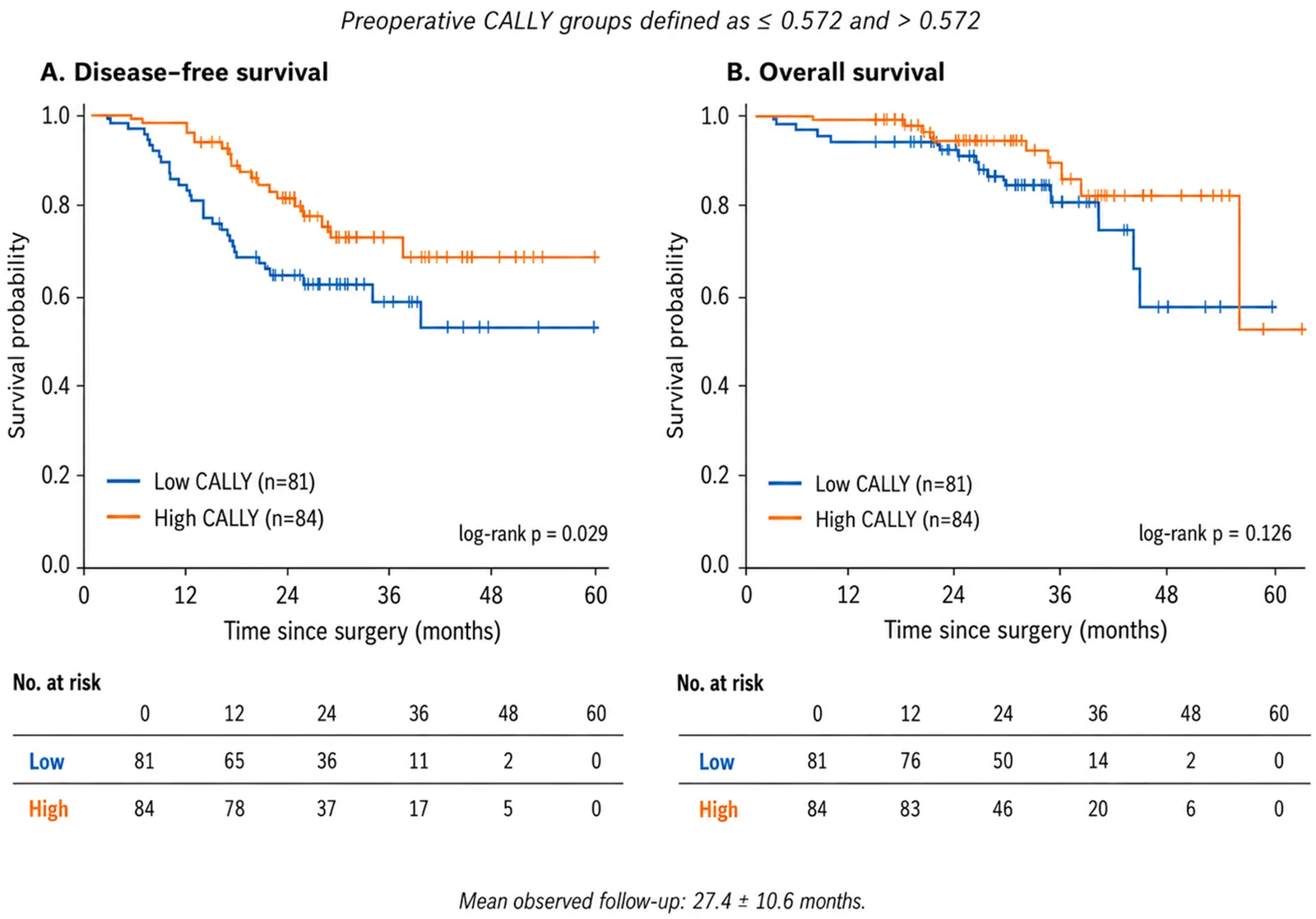
Kaplan–Meier survival curves in the follow-up-mature cohort according to preoperative CALLY group. Disease-free survival was measured from curative gastrectomy to recurrence, death from any cause, or last follow-up **(A)**. Overall survival was measured from curative gastrectomy to death from any cause or last follow-up **(B)**. Low CALLY was defined as preoperative CALLY ≤0.572, and high CALLY as preoperative CALLY >0.572. Patients with low preoperative CALLY had worse DFS than those with high CALLY (log-rank p = 0.029), whereas OS did not differ significantly (log-rank p = 0.126). The mean observed follow-up was 27.4 ± 10.6 months. Group-specific number-at-risk tables at 12-month intervals are shown below the curves, and censor marks indicate censored observations. CALLY, C-reactive protein–albumin–lymphocyte index; DFS, disease-free survival; OS, overall survival.

Survival outcomes are summarized in **Table 4** and illustrated in **Figure 4**.

**Table 4:** Survival outcomes in the follow-up-mature cohort.

A. Events and Kaplan–Meier estimates				
Outcome	Overall (n=165)	Low CALLY (n=81)	High CALLY (n=84)	Overall comparison p value
Documented recurrence	41 (24.8)	24 (29.6)	17 (20.2)	—
DFS event	52 (31.5)	32 (39.5)	20 (23.8)	—
Death	24 (14.5)	15 (18.5)	9 (10.7)	—
1-year DFS	86.7%	80.2%	92.9%	
2-year DFS	69.3%	62.1%	76.3%	
3-year DFS	63.5%	58.6%	67.9%	
DFS log-rank	—	—	—	0.029
1-year OS	96.4%	93.8%	98.8%	
2-year OS	90.9%	87.5%	94.4%	
3-year OS	80.6%	80.2%	81.5%	
OS log-rank	—	—	—	0.126
B. Cox regression for DFS				
CALLY variable		Hazard ratio (95% CI)		p value
Low versus high CALLY, univariable		1.85 (1.06–3.23)		0.032
Continuous CALLY, univariable		0.51 (0.29–0.91)		0.022
Continuous CALLY, multivariable		0.58 (0.33–1.04)		0.067

The follow-up-mature cohort included patients who underwent surgery on or before 31 May 2025. The full-cohort CALLY threshold of 0.572 was retained. Mean observed follow-up was 834.8 ± 322.2 days (27.4 ± 10.6 months). DFS was measured from surgery to recurrence, death, or last follow-up; OS was measured from surgery to death or last follow-up. The multivariable DFS model was adjusted for stage III disease, lymphovascular invasion, perineural invasion, and major postoperative complications. Categorical and continuous CALLY variables were evaluated in separate Cox models and were not entered simultaneously. The multivariable model used continuous preoperative CALLY. P values were not calculated for crude event counts or individual annual Kaplan–Meier estimates because these summaries do not independently account for censoring and were not prespecified as separate hypothesis tests. Between-group survival comparisons were based on the log-rank test over the complete follow-up period, and hazard ratios were estimated using Cox regression.

Low-CALLY patients had worse DFS than high-CALLY patients (log-rank p=0.029). The 1-, 2-, and 3-year DFS estimates were 80.2%, 62.1%, and 58.6% in the low-CALLY group and 92.9%, 76.3%, and 67.9% in the high-CALLY group, respectively. Low CALLY was associated with DFS in univariable Cox analysis (HR, 1.85; 95% CI, 1.06–3.23; p=0.032). Continuous CALLY was also associated with DFS in univariable analysis (HR, 0.51; 95% CI, 0.29–0.91; p=0.022), although this association was attenuated after adjustment for stage III disease, lymphovascular invasion, perineural invasion, and major complications (adjusted HR, 0.58; 95% CI, 0.33–1.04; p=0.067).

OS did not differ significantly between CALLY groups (log-rank p=0.126). Complete-cohort and observed-follow-up ≥12-month sensitivity analyses were directionally consistent and are presented in **Supplementary Table 8** and **Supplementary Figure 2**.

## Discussion

This study identifies three complementary dimensions of perioperative CALLY assessment after curative gastrectomy for gastric adenocarcinoma. First, low preoperative CALLY was associated with greater quantitative nodal tumor burden and remained independently associated with high LNR and high LODDS. Second, patient-normalized postoperative CALLY recovery differed markedly according to major complication and textbook surgical outcome status. Third, low CALLY was associated with inferior DFS in a follow-up-mature cohort, although the continuous association was attenuated after multivariable adjustment. These findings support a distinction between preoperative CALLY as a marker of tumor–host biology and postoperative CALLY dynamics as a marker of recovery.

Nodal status remains a major prognostic determinant in gastric cancer, but conventional pN classification depends on the absolute number of metastatic nodes and may be influenced by nodal retrieval. LNR and LODDS provide complementary quantitative estimates of lymphatic dissemination (5–7). The independent relationships between low CALLY and high LNR and high LODDS extend previous CALLY studies, which have predominantly focused on survival endpoints (8–13). This association is biologically plausible because cancer-related inflammation may facilitate tumor progression, immune escape, and metastatic dissemination, whereas hypoalbuminemia and lymphopenia may reflect impaired nutritional and cellular immune reserve (20–23). The findings suggest that systemic inflammation, nutritional reserve, and cellular immune competence may parallel the biological intensity of nodal spread, although the retrospective design does not permit causal interpretation.

To ensure adequate follow-up maturity, primary survival analyses were restricted to patients with at least 12 months of potential observation based on the calendar date of surgery.

Low CALLY remained associated with worse DFS by Kaplan–Meier and univariable Cox analysis. However, the association of continuous CALLY was no longer statistically significant after multivariable adjustment, despite a consistent protective direction. Complete-cohort and strict observed-follow-up sensitivity analyses produced directionally similar estimates. Accordingly, the present findings support a clinically relevant DFS signal but do not establish CALLY as an independently validated survival biomarker.

The longitudinal analysis provides more direct evidence for the dynamic component of CALLY. By normalizing postoperative values to each patient’s preoperative baseline, the recovery ratio reduced between-patient differences in absolute CALLY and quantified the relative postoperative trajectory. Patients with major complications and those who failed textbook surgical outcome demonstrated deeper and more persistent suppression. These findings are consistent with reports linking postoperative CRP-based indices and NLR with adverse outcomes after gastrectomy (14, 15). The component-level analyses further qualified the clinical interpretation of CALLY. Although preoperative CALLY showed numerically greater discrimination than CRP, albumin, or lymphocyte count for the selected nodal and DFS endpoints, its advantage over CRP and albumin was not statistically significant. In the early postoperative period, CRP equaled or exceeded the discriminatory performance of CALLY, whereas CALLY demonstrated higher discrimination at discharge. CALLY should therefore not be considered a universally superior replacement for its constituent laboratory parameters. Its potential value may instead lie in providing an integrated representation of inflammatory, nutritional, and immune recovery later in the postoperative course.

The temporal interpretation of postoperative CALLY requires caution. Because 34 of 39 major complications in the open-gastrectomy cohort had been documented by POD7, POD7 and discharge CALLY should be regarded as monitoring markers of an evolving or established adverse postoperative course rather than prospective predictive biomarkers. POD1 CALLY was obtained before the documented onset of major complications and may provide earlier monitoring information than later measurements; however, prospective validation with standardized sampling and complication adjudication remains necessary.

The adapted textbook surgical outcome definition incorporated a literature-based LOS threshold of <21 days to improve comparability with published gastrectomy frameworks (16, 17, 24–26). Postoperative morbidity after gastrectomy is influenced by case mix and procedural complexity and remains clinically consequential, as demonstrated by institutional series, national audits, international benchmark studies, and long-term survival analyses (27–30). Restricting primary postoperative analyses to open gastrectomy also reduced potential confounding by surgical approach; complete-cohort sensitivity analyses produced similar findings.

This study has several strengths, including a homogeneous curative-intent cohort, verification of patient-specific diagnosis, surgery, recurrence, death, and follow-up dates, evaluation of multiple complementary nodal metrics, and integration of patient-normalized longitudinal biomarker analysis with surgical and oncological outcomes. Nevertheless, the study is retrospective and single-center. Because eligibility was restricted to R0, non-metastatic resections, the findings may not be generalizable to R1/R2 resections or metastatic and palliative settings. CALLY may be influenced by non-cancer inflammatory conditions, comorbidity, nutritional interventions, and body composition, which were not fully captured. Discharge measurements were obtained at variable postoperative intervals and should not be interpreted as fixed-time biomarker assessments. The full-cohort median CALLY threshold and adapted textbook surgical outcome definition require external validation. Postoperative and component-level ROC analyses were exploratory, were not adjusted for multiple comparisons, and quantify relative discrimination rather than incremental clinical utility, net benefit, or cost-effectiveness. Finally, the number of death events was limited, and the adjusted DFS association did not meet the conventional threshold for statistical significance in the primary follow-up-mature cohort.

Taken together, preoperative CALLY was associated with quantitative nodal tumor burden, whereas relative postoperative CALLY recovery reflected the quality of the postoperative course. These complementary findings justify external multicenter validation but do not support the use of CALLY as a stand-alone decision-making or predictive tool at present.

## Conclusion

Low preoperative CALLY was independently associated with high LNR and high LODDS after curative gastrectomy for gastric adenocarcinoma. Impaired within-patient postoperative CALLY recovery was associated with major complications and failure to achieve textbook surgical outcome. Low CALLY was also associated with worse DFS in the follow-up-mature cohort, although the continuous association was attenuated after multivariable adjustment. Postoperative CALLY should be interpreted as a monitoring index rather than a stand-alone predictive biomarker, and its relative value compared with individual components—particularly CRP—appears to be time-dependent. External multicenter validation is required before clinical implementation.

## Data Availability

The datasets presented in this article are not readily available because the data supporting the findings of this study are available from the corresponding author upon reasonable request. The data are not publicly available due to patient privacy considerations, ethical restrictions, and institutional data protection policies. Requests to access the datasets should be directed to dr.ademozcan@gmail.com.

## Supplementary material

Supplementary Figure 1 Exploratory receiver operating characteristic analyses of preoperative inflammatory–nutritional indices and postoperative CALLY values. **(A)** Preoperative CALLY was evaluated for pN-positive disease and high lymph node ratio in the complete curative-intent cohort and for DFS events in the follow-up-mature cohort. **(B)** Preoperative CALLY, PNI, NLR, PLR, and mGPS were compared for discrimination of DFS events in the follow-up-mature cohort. **(C)** Postoperative CALLY values were evaluated for major postoperative complications in the open-gastrectomy cohort. **(D)** Postoperative CALLY values were evaluated for failure to achieve the adapted textbook surgical outcome in the open-gastrectomy cohort. For CALLY and PNI, lower values indicated higher-risk status; inverse marker values were therefore used when constructing the ROC curves. The analyses were exploratory. The postoperative ROC panels quantify the concurrent discrimination of monitoring markers and should not be interpreted as prospective prediction or diagnostic validation. AUC values are shown in the legends. CALLY, C-reactive protein–albumin–lymphocyte index; PNI, Prognostic Nutritional Index; NLR, neutrophil-to-lymphocyte ratio; PLR, platelet-to-lymphocyte ratio; mGPS, modified Glasgow Prognostic Score; LNR, lymph node ratio; DFS, disease-free survival; POD, postoperative day; ROC, receiver operating characteristic; AUC, area under the curve.

Supplementary Figure 2 Complete-cohort Kaplan–Meier sensitivity analyses according to preoperative CALLY group. Disease-free survival was measured from curative gastrectomy to documented recurrence, death from any cause, or last follow-up **(A)**. Overall survival was measured from curative gastrectomy to death from any cause or last follow-up **(B)**. Low CALLY was defined as preoperative CALLY ≤0.572, and high CALLY as CALLY >0.572. In the complete curative-intent cohort of 188 patients, low CALLY was associated with worse DFS (log-rank p=0.012), whereas OS did not differ significantly between groups (log-rank p=0.134). Number-at-risk tables are shown below the curves, and vertical marks indicate censored observations. CALLY, C-reactive protein–albumin–lymphocyte index; DFS, disease-free survival; OS, overall survival.
