# Supplementary material for: Perioperative CALLY dynamics after curative gastrectomy for gastric adenocarcinoma: associations with nodal tumor burden, textbook surgical outcome, and disease-free survival
Source: Front Oncol. 2026 Sep 3;16:1915467. doi: 10.3389/fonc.2026.1915467 (PMC13581691; doi:10.3389/fonc.2026.1915467)
Supplement: Supplementary file 1 [file SupplementaryFile1.zip › Supplementary Table S1.docx]

**Supplementary Table S1. Complete baseline characteristics according to preoperative CALLY group**

| **Variable** | **Overall (n=188)** | **Low CALLY (n=94)** | **High CALLY (n=94)** | **p value** |
| --- | --- | --- | --- | --- |
| **Demographic and clinical variables** | | | | |
| Age, years | 67.0 (57.0–74.3) | 67.5 (55.3–75.0) | 67.0 (57.3–73.8) | 0.874 |
| **Sex** |  |  |  | 0.550 |
| Female | 74 (39.4) | 35 (37.2) | 39 (41.5) |  |
| Male | 114 (60.6) | 59 (62.8) | 55 (58.5) |  |
| ASA III–IV | 99 (52.7) | 51 (54.3) | 48 (51.1) | 0.661 |
| ECOG performance status ≥1 | 143 (76.1) | 74 (78.7) | 69 (73.4) | 0.393 |
| **Treatment and surgical variables** | | | | |
| Neoadjuvant chemotherapy | 36 (19.1) | 17 (18.1) | 19 (20.2) | 0.711 |
| **Neoadjuvant regimen** |  |  |  | 0.901 |
| Not administered | 152 (80.9) | 77 (81.9) | 75 (79.8) |  |
| CAPOX/XELOX | 14 (7.4) | 7 (7.4) | 7 (7.4) |  |
| FLOT | 22 (11.7) | 10 (10.6) | 12 (12.8) |  |
| **Type of gastrectomy** |  |  |  | 0.050 |
| Subtotal distal gastrectomy | 90 (47.9) | 49 (52.1) | 41 (43.6) |  |
| Total gastrectomy | 86 (45.7) | 36 (38.3) | 50 (53.2) |  |
| Proximal gastrectomy | 12 (6.4) | 9 (9.6) | 3 (3.2) |  |
| **Surgical approach** |  |  |  | 0.194 |
| Open | 178 (94.7) | 91 (96.8) | 87 (92.6) |  |
| Laparoscopic | 10 (5.3) | 3 (3.2) | 7 (7.4) |  |
| **Preoperative laboratory variables and indices** | | | | |
| CEA, ng/mL | 3.0 (1.6–5.3) | 2.8 (1.6–5.9) | 3.3 (1.7–5.0) | 0.920 |
| CA 19-9, U/mL | 20.1 (9.6–36.5) | 18.8 (8.9–31.8) | 21.2 (10.8–40.9) | 0.279 |
| Albumin, g/dL | 3.9 (3.7–4.2) | 3.8 (3.6–4.1) | 4.0 (3.7–4.3) | 0.003 |
| CRP, mg/L | 9.6 (6.3–14.4) | 14.3 (11.6–18.5) | 6.4 (4.6–8.4) | <0.001 |
| Neutrophil count, ×10⁹/L | 4.82 (3.94–5.84) | 5.19 (4.26–5.89) | 4.62 (3.63–5.71) | 0.033 |
| Lymphocyte count, ×10⁹/L | 1.54 (1.25–1.98) | 1.29 (1.10–1.56) | 1.86 (1.53–2.23) | <0.001 |
| Platelet count, ×10⁹/L | 278.5 (237.0–331.0) | 292.5 (249.5–344.8) | 265.0 (223.0–296.2) | 0.002 |
| Hemoglobin, g/dL | 11.6 (10.8–12.7) | 11.7 (10.6–12.6) | 11.6 (10.8–13.1) | 0.542 |
| Preoperative CALLY | 0.572 (0.379–1.191) | 0.379 (0.259–0.485) | 1.192 (0.779–1.571) | <0.001 |
| NLR | 3.078 (2.356–4.195) | 3.856 (3.072–5.149) | 2.583 (1.957–3.084) | <0.001 |
| PLR | 172.6 (131.0–239.9) | 227.0 (170.0–292.1) | 142.7 (108.1–177.2) | <0.001 |
| PNI | 47.1 (44.0–50.3) | 45.4 (41.8–47.6) | 49.5 (46.8–53.0) | <0.001 |
| **mGPS** |  |  |  | <0.001 |
| 0 | 99 (52.7) | 16 (17.0) | 83 (88.3) |  |
| 1 | 76 (40.4) | 66 (70.2) | 10 (10.6) |  |
| 2 | 13 (6.9) | 12 (12.8) | 1 (1.1) |  |

**Note:** Data are presented as median (interquartile range) or n (%). Low and high CALLY groups were defined using the full-cohort median preoperative CALLY value of 0.572. Because albumin, CRP, and lymphocyte count are components of CALLY, their between-group p values are descriptive and should not be interpreted as independent associations. ASA, American Society of Anesthesiologists; ECOG, Eastern Cooperative Oncology Group; CALLY, C-reactive protein–albumin–lymphocyte index; CRP, C-reactive protein; NLR, neutrophil-to-lymphocyte ratio; PLR, platelet-to-lymphocyte ratio; PNI, Prognostic Nutritional Index; mGPS, modified Glasgow Prognostic Score; CAPOX/XELOX, capecitabine plus oxaliplatin; FLOT, fluorouracil/leucovorin, oxaliplatin, and docetaxel.
