# Supplementary material for: Perioperative CALLY dynamics after curative gastrectomy for gastric adenocarcinoma: associations with nodal tumor burden, textbook surgical outcome, and disease-free survival
Source: Front Oncol. 2026 Sep 3;16:1915467. doi: 10.3389/fonc.2026.1915467 (PMC13581691; doi:10.3389/fonc.2026.1915467)
Supplement: Supplementary file 1 [file SupplementaryFile1.zip › Supplementary Table S2.docx]

**Supplementary Table S2. Complete pathological characteristics and nodal tumor burden according to preoperative CALLY group**

| **Variable** | **Overall (n=188)** | **Low CALLY (n=94)** | **High CALLY (n=94)** | **p value** |
| --- | --- | --- | --- | --- |
| **Tumor characteristics** | | | | |
| Tumor size, mm | 49.5 (33.0–63.3) | 56.0 (42.3–74.5) | 42.5 (30.0–53.0) | <0.001 |
| **Lauren classification** |  |  |  | 0.484 |
| Intestinal | 91 (48.4) | 46 (48.9) | 45 (47.9) |  |
| Diffuse | 70 (37.2) | 32 (34.0) | 38 (40.4) |  |
| Mixed | 27 (14.4) | 16 (17.0) | 11 (11.7) |  |
| **Histological grade** |  |  |  | 0.585 |
| G1 (well differentiated) | 4 (2.1) | 2 (2.1) | 2 (2.1) |  |
| G2 (moderately differentiated) | 85 (45.2) | 39 (41.5) | 46 (48.9) |  |
| G3 (poorly differentiated) | 99 (52.7) | 53 (56.4) | 46 (48.9) |  |
| Linitis plastica | 18 (9.6) | 7 (7.4) | 11 (11.7) | 0.457 |
| **Aggressive pathological features** | | | | |
| Lymphovascular invasion | 68 (36.2) | 40 (42.6) | 28 (29.8) | 0.095 |
| Perineural invasion | 77 (41.0) | 44 (46.8) | 33 (35.1) | 0.138 |
| Serosal invasion | 41 (21.8) | 38 (40.4) | 3 (3.2) | <0.001 |
| **Pathological T stage** |  |  |  | <0.001 |
| T1 | 25 (13.3) | 9 (9.6) | 16 (17.0) |  |
| T2 | 38 (20.2) | 10 (10.6) | 28 (29.8) |  |
| T3 | 84 (44.7) | 37 (39.4) | 47 (50.0) |  |
| T4 | 41 (21.8) | 38 (40.4) | 3 (3.2) |  |
| **Pathological N stage** |  |  |  | 0.004 |
| N0 | 61 (32.4) | 21 (22.3) | 40 (42.6) |  |
| N1 | 58 (30.9) | 28 (29.8) | 30 (31.9) |  |
| N2 | 46 (24.5) | 32 (34.0) | 14 (14.9) |  |
| N3 | 23 (12.2) | 13 (13.8) | 10 (10.6) |  |
| **AJCC eighth-edition pathological stage** |  |  |  | <0.001 |
| IA | 19 (10.1) | 6 (6.4) | 13 (13.8) |  |
| IB | 27 (14.4) | 11 (11.7) | 16 (17.0) |  |
| IIA | 30 (16.0) | 6 (6.4) | 24 (25.5) |  |
| IIB | 34 (18.1) | 17 (18.1) | 17 (18.1) |  |
| IIIA | 55 (29.3) | 41 (43.6) | 14 (14.9) |  |
| IIIB | 18 (9.6) | 9 (9.6) | 9 (9.6) |  |
| IIIC | 5 (2.7) | 4 (4.3) | 1 (1.1) |  |
| **Nodal tumor burden** | | | | |
| Retrieved lymph nodes | 31 (26–35) | 31 (26–35) | 31 (26–35) | 0.624 |
| Metastatic lymph nodes | 2 (0–4) | 2 (1–5) | 1 (0–3.5) | 0.001 |
| pN-positive disease | 127 (67.6) | 73 (77.7) | 54 (57.4) | 0.005 |
| N3 disease | 23 (12.2) | 13 (13.8) | 10 (10.6) | 0.656 |
| Lymph node ratio | 0.053 (0–0.154) | 0.077 (0.027–0.194) | 0.029 (0–0.119) | 0.002 |
| LODDS | −2.605 (−3.980 to −1.609) | −2.282 (−3.205 to −1.371) | −3.106 (−4.111 to −1.897) | 0.005 |

**Note:** Data are presented as median (interquartile range) or n (%). AJCC, American Joint Committee on Cancer; CALLY, C-reactive protein–albumin–lymphocyte index; LNR, lymph node ratio; LODDS, log odds of positive lymph nodes.
