# Supplementary material for: Perioperative CALLY dynamics after curative gastrectomy for gastric adenocarcinoma: associations with nodal tumor burden, textbook surgical outcome, and disease-free survival
Source: Front Oncol. 2026 Sep 3;16:1915467. doi: 10.3389/fonc.2026.1915467 (PMC13581691; doi:10.3389/fonc.2026.1915467)
Supplement: Supplementary file 1 [file SupplementaryFile1.zip › Supplementary Table S3.docx]

**Supplementary Table S3. Absolute perioperative CALLY values and postoperative outcomes**

**Panel A. Absolute CALLY values according to major postoperative complication status in the open-gastrectomy cohort**

| **Time point** | **No major complication (n=139)** | **Major complication (n=39)** | **p value** |
| --- | --- | --- | --- |
| Preoperative CALLY | 0.5668 (0.3723–1.2467) | 0.5490 (0.3889–0.8673) | 0.452 |
| POD1 CALLY | 0.0393 (0.0296–0.0520) | 0.0190 (0.0120–0.0274) | <0.001 |
| POD3 CALLY | 0.0250 (0.0169–0.0365) | 0.0066 (0.0040–0.0103) | <0.001 |
| POD7 CALLY | 0.0662 (0.0403–0.0948) | 0.0072 (0.0046–0.0130) | <0.001 |
| Discharge CALLY | 0.0932 (0.0636–0.1438) | 0.0122 (0.0060–0.0246) | <0.001 |

**Panel B. Absolute CALLY values according to adapted textbook surgical outcome status in the open-gastrectomy cohort**

| **Time point** | **Achieved (n=130)** | **Not achieved (n=48)** | **p value** |
| --- | --- | --- | --- |
| Preoperative CALLY | 0.5842 (0.3822–1.2570) | 0.5298 (0.3757–0.8302) | 0.221 |
| POD1 CALLY | 0.0398 (0.0301–0.0523) | 0.0207 (0.0127–0.0297) | <0.001 |
| POD3 CALLY | 0.0255 (0.0174–0.0369) | 0.0074 (0.0043–0.0116) | <0.001 |
| POD7 CALLY | 0.0690 (0.0459–0.0973) | 0.0085 (0.0056–0.0162) | <0.001 |
| Discharge CALLY | 0.0977 (0.0670–0.1467) | 0.0162 (0.0065–0.0359) | <0.001 |

**Panel C. Overall postoperative outcomes in the complete cohort**

| **Outcome** | **Overall (n=188)** |
| --- | --- |
| ICU admission | 60 (31.9) |
| Anastomotic leakage | 10 (5.3) |
| Intra-abdominal abscess | 39 (20.7) |
| Surgical site infection | 43 (22.9) |
| Sepsis | 26 (13.8) |
| 30-day reoperation | 15 (8.0) |
| 30-day readmission | 11 (5.9) |
| 30-day mortality | 0 (0.0) |
| 90-day mortality | 2 (1.1) |
| Length of stay, days | 9.0 (8.0–11.0) |
| Major postoperative complication | 40 (21.3) |
| Adapted textbook surgical outcome achieved | 139 (73.9) |

Data are presented as median (interquartile range) or n (%). Time-point p values in Panels A and B are descriptive and unadjusted; primary longitudinal inference was based on the time-by-group interaction in the generalized estimating equation models. The adapted textbook surgical outcome incorporated a postoperative length-of-stay threshold of <21 days. CALLY, C-reactive protein–albumin–lymphocyte index; ICU, intensive care unit; POD, postoperative day.
