# Supplementary material for: Perioperative CALLY dynamics after curative gastrectomy for gastric adenocarcinoma: associations with nodal tumor burden, textbook surgical outcome, and disease-free survival
Source: Front Oncol. 2026 Sep 3;16:1915467. doi: 10.3389/fonc.2026.1915467 (PMC13581691; doi:10.3389/fonc.2026.1915467)
Supplement: Supplementary file 1 [file SupplementaryFile1.zip › Supplementary Table S4.docx]

**Supplementary Table S4. Exploratory receiver operating characteristic analyses**

**Panel A. Preoperative indices for pN-positive disease (127 events/188 patients)**

| **Marker** | **AUC (95% CI)** | **Optimal cutoff** | **Sensitivity, %** | **Specificity, %** | **p value** |
| --- | --- | --- | --- | --- | --- |
| CALLY | 0.613 (0.528–0.693) | ≤0.5906 | 59.8 | 65.6 | 0.012 |
| PNI | 0.590 (0.487–0.667) | ≤46.85 | 55.1 | 67.2 | 0.047 |
| NLR | 0.544 (0.452–0.640) | ≥3.07 | 55.1 | 57.4 | 0.326 |
| PLR | 0.529 (0.446–0.612) | ≥298.50 | 15.7 | 93.4 | 0.528 |
| mGPS | 0.630 (0.552–0.702) | ≥1 | 55.1 | 68.9 | 0.001 |

**Panel B. Preoperative indices for high LNR (93 events/188 patients)**

| **Marker** | **AUC (95% CI)** | **Optimal cutoff** | **Sensitivity, %** | **Specificity, %** | **p value** |
| --- | --- | --- | --- | --- | --- |
| CALLY | 0.631 (0.547–0.711) | ≤0.5778 | 65.6 | 63.2 | 0.002 |
| PNI | 0.604 (0.524–0.678) | ≤48.05 | 72.0 | 50.5 | 0.014 |
| NLR | 0.534 (0.453–0.621) | ≥4.31 | 26.9 | 82.1 | 0.421 |
| PLR | 0.578 (0.498–0.662) | ≥174.66 | 58.1 | 58.9 | 0.065 |
| mGPS | 0.632 (0.556–0.702) | ≥1 | 59.1 | 64.2 | <0.001 |

**Panel C. Preoperative indices for DFS events in the follow-up-mature cohort (52 events/165 patients)**

| **Marker** | **AUC (95% CI)** | **Optimal cutoff** | **Sensitivity, %** | **Specificity, %** | **p value** |
| --- | --- | --- | --- | --- | --- |
| CALLY | 0.631 (0.544–0.716) | ≤0.7076 | 76.9 | 51.3 | 0.007 |
| PNI | 0.573 (0.471–0.674) | ≤45.60 | 50.0 | 69.0 | 0.133 |
| NLR | 0.572 (0.476–0.668) | ≥3.39 | 51.9 | 64.6 | 0.137 |
| PLR | 0.619 (0.520–0.702) | ≥197.14 | 57.7 | 67.3 | 0.014 |
| mGPS | 0.584 (0.500–0.681) | ≥1 | 57.7 | 59.3 | 0.049 |

**Panel D. Postoperative CALLY for major complications in the open-gastrectomy cohort (39 events/178 patients)**

| **Marker** | **AUC (95% CI)** | **Optimal cutoff** | **Sensitivity, %** | **Specificity, %** | **p value** |
| --- | --- | --- | --- | --- | --- |
| POD1 CALLY | 0.849 (0.785–0.907) | ≤0.0292 | 82.1 | 77.7 | <0.001 |
| POD3 CALLY | 0.951 (0.919–0.978) | ≤0.0111 | 84.6 | 92.1 | <0.001 |
| POD7 CALLY | 0.987 (0.977–0.997) | ≤0.0236 | 97.4 | 92.8 | <0.001 |
| Discharge CALLY | 0.958 (0.926–0.984) | ≤0.0367 | 87.2 | 90.6 | <0.001 |

**Panel E. Postoperative CALLY for adapted textbook surgical outcome failure in the open-gastrectomy cohort (48 events/178 patients)**

| **Marker** | **AUC (95% CI)** | **Optimal cutoff** | **Sensitivity, %** | **Specificity, %** | **p value** |
| --- | --- | --- | --- | --- | --- |
| POD1 CALLY | 0.832 (0.760–0.890) | ≤0.0292 | 75.0 | 79.2 | <0.001 |
| POD3 CALLY | 0.930 (0.885–0.966) | ≤0.0163 | 91.7 | 79.2 | <0.001 |
| POD7 CALLY | 0.967 (0.940–0.988) | ≤0.0236 | 85.4 | 94.6 | <0.001 |
| Discharge CALLY | 0.945 (0.910–0.974) | ≤0.0430 | 83.3 | 90.8 | <0.001 |

High LNR was defined as LNR above the exact full-cohort median (0.0526316; reported as 0.0526). Optimal cutoffs were selected using the Youden index. AUC confidence intervals were estimated using nonparametric bootstrap resampling. For CALLY and PNI, lower values indicate higher-risk status; for NLR, PLR, and mGPS, higher values indicate higher-risk status. Postoperative ROC analyses were restricted to the open-gastrectomy cohort and should be interpreted as exploratory discrimination analyses rather than prospective diagnostic validation. AUC, area under the receiver operating characteristic curve; CI, confidence interval; DFS, disease-free survival; LNR, lymph node ratio; POD, postoperative day; ROC, receiver operating characteristic.
