# Supplementary material for: Perioperative CALLY dynamics after curative gastrectomy for gastric adenocarcinoma: associations with nodal tumor burden, textbook surgical outcome, and disease-free survival
Source: Front Oncol. 2026 Sep 3;16:1915467. doi: 10.3389/fonc.2026.1915467 (PMC13581691; doi:10.3389/fonc.2026.1915467)
Supplement: Supplementary file 1 [file SupplementaryFile1.zip › Supplementary Table S5.docx]

**Supplementary Table S5. Full univariable and multivariable regression models for nodal outcomes and disease-free survival**

**Panel A. pN-positive disease - logistic regression (127 events/188 patients)**

| **Variable** | **Univariable estimate (95% CI)** | **p value** | **Adjusted estimate (95% CI)** | **p value** |
| --- | --- | --- | --- | --- |
| Low CALLY (≤0.572) | 2.57 (1.37-4.86) | 0.003 | 1.62 (0.77-3.39) | 0.200 |
| Age, per year | 1.01 (0.98-1.03) | 0.588 | 1.02 (0.99-1.05) | 0.176 |
| Male sex | 1.35 (0.73-2.51) | 0.341 | 1.15 (0.55-2.40) | 0.717 |
| Neoadjuvant chemotherapy | 1.87 (0.80-4.40) | 0.149 | 1.70 (0.65-4.45) | 0.282 |
| Pathological T3-T4 disease | 9.78 (4.83-19.80) | <0.001 | 9.02 (4.16-19.56) | <0.001 |
| Lymphovascular invasion | 1.74 (0.90-3.37) | 0.103 | 0.99 (0.45-2.18) | 0.978 |
| Perineural invasion | 1.67 (0.88-3.16) | 0.116 | 1.13 (0.53-2.39) | 0.757 |

**Panel B. High lymph node ratio - logistic regression (93 events/188 patients)**

| **Variable** | **Univariable estimate (95% CI)** | **p value** | **Adjusted estimate (95% CI)** | **p value** |
| --- | --- | --- | --- | --- |
| Low CALLY (≤0.572) | 2.97 (1.64-5.38) | <0.001 | 2.03 (1.04-3.97) | 0.037 |
| Age, per year | 1.00 (0.98-1.03) | 0.865 | 1.01 (0.98-1.04) | 0.451 |
| Male sex | 1.06 (0.59-1.90) | 0.856 | 0.88 (0.44-1.75) | 0.711 |
| Neoadjuvant chemotherapy | 0.78 (0.38-1.62) | 0.503 | 0.59 (0.26-1.34) | 0.205 |
| Pathological T3-T4 disease | 9.01 (4.27-19.04) | <0.001 | 8.33 (3.71-18.70) | <0.001 |
| Lymphovascular invasion | 2.19 (1.19-4.02) | 0.012 | 1.50 (0.75-3.00) | 0.248 |
| Perineural invasion | 1.29 (0.72-2.31) | 0.388 | 0.77 (0.39-1.54) | 0.465 |

**Panel C. High LODDS - logistic regression (94 events/188 patients)**

| **Variable** | **Univariable estimate (95% CI)** | **p value** | **Adjusted estimate (95% CI)** | **p value** |
| --- | --- | --- | --- | --- |
| Low CALLY (≤0.572) | 3.11 (1.72-5.65) | <0.001 | 2.11 (1.08-4.14) | 0.029 |
| Age, per year | 1.00 (0.97-1.02) | 0.865 | 1.00 (0.98-1.03) | 0.726 |
| Male sex | 1.09 (0.61-1.96) | 0.765 | 0.94 (0.47-1.87) | 0.850 |
| Neoadjuvant chemotherapy | 0.76 (0.37-1.58) | 0.459 | 0.56 (0.24-1.29) | 0.171 |
| Pathological T3-T4 disease | 9.34 (4.42-19.76) | <0.001 | 8.28 (3.70-18.55) | <0.001 |
| Lymphovascular invasion | 2.33 (1.26-4.29) | 0.007 | 1.62 (0.81-3.24) | 0.175 |
| Perineural invasion | 1.36 (0.76-2.44) | 0.300 | 0.81 (0.41-1.62) | 0.554 |

**Panel D. Disease-free survival - Cox regression (52 events/165 patients)**

| **Variable** | **Univariable estimate (95% CI)** | **p value** | **Adjusted estimate (95% CI)** | **p value** |
| --- | --- | --- | --- | --- |
| Low CALLY (≤0.572) | 1.85 (1.06-3.23) | 0.032 | - | - |
| Preoperative CALLY, continuous | 0.51 (0.29-0.91) | 0.022 | 0.58 (0.33-1.04) | 0.067 |
| Stage III disease | 1.96 (1.13-3.40) | 0.016 | 1.70 (0.95-3.06) | 0.075 |
| Lymphovascular invasion | 1.00 (0.57-1.76) | 0.997 | 0.91 (0.50-1.63) | 0.742 |
| Perineural invasion | 1.13 (0.65-1.95) | 0.666 | 0.96 (0.55-1.68) | 0.888 |
| Major postoperative complication | 0.71 (0.34-1.52) | 0.379 | 0.66 (0.31-1.42) | 0.287 |

**Note:** Panels A-C report odds ratios from logistic regression; Panel D reports hazard ratios from Cox regression. High LNR and high LODDS were defined using the full-cohort medians (LNR > 0.0526316, displayed as > 0.0526; LODDS > -2.605107, displayed as > -2.605). The multivariable logistic models included all variables shown in the corresponding panels. The multivariable Cox model included continuous preoperative CALLY, stage III disease, lymphovascular invasion, perineural invasion, and major postoperative complications in the follow-up-mature cohort; ties were handled using the Efron method. Dichotomized and continuous CALLY were not entered simultaneously in the Cox model; therefore, no adjusted estimate is shown for low versus high CALLY. Reference categories were high CALLY, female sex, no neoadjuvant chemotherapy, pathological T1-T2 disease, and absence of lymphovascular or perineural invasion. OR, odds ratio; HR, hazard ratio; CI, confidence interval; LNR, lymph node ratio; LODDS, log odds of positive lymph nodes; CALLY, C-reactive protein-albumin-lymphocyte index.
