# Supplementary material for: Perioperative CALLY dynamics after curative gastrectomy for gastric adenocarcinoma: associations with nodal tumor burden, textbook surgical outcome, and disease-free survival
Source: Front Oncol. 2026 Sep 3;16:1915467. doi: 10.3389/fonc.2026.1915467 (PMC13581691; doi:10.3389/fonc.2026.1915467)
Supplement: Supplementary file 1 [file SupplementaryFile1.zip › Supplementary Table S6.docx]

**Supplementary Table S6. Complete-cohort longitudinal and ROC sensitivity analyses**

**Panel A. CALLY recovery ratios according to major postoperative complication status**

| **Time point** | **No major complication (n=148)** | **Major complication (n=40)** |
| --- | --- | --- |
| POD1 | 0.0608 (0.0408-0.0934) | 0.0302 (0.0224-0.0461) |
| POD3 | 0.0373 (0.0257-0.0565) | 0.0102 (0.0073-0.0156) |
| POD7 | 0.0946 (0.0593-0.1501) | 0.0117 (0.0076-0.0183) |
| Discharge | 0.1521 (0.0881-0.2342) | 0.0186 (0.0120-0.0492) |

**Time-by-group interaction: Wald χ²(3)=833.8; p<0.001.**

**Panel B. CALLY recovery ratios according to adapted textbook surgical outcome status**

| **Time point** | **Achieved (n=139)** | **Not achieved (n=49)** |
| --- | --- | --- |
| POD1 | 0.0611 (0.0408-0.0944) | 0.0332 (0.0239-0.0503) |
| POD3 | 0.0378 (0.0268-0.0605) | 0.0125 (0.0077-0.0221) |
| POD7 | 0.1011 (0.0625-0.1615) | 0.0156 (0.0082-0.0306) |
| Discharge | 0.1595 (0.0927-0.2423) | 0.0238 (0.0136-0.0597) |

**Time-by-group interaction: Wald χ²(3)=364.4; p<0.001.**

**Panel C. Exploratory ROC analyses in the complete cohort**

| **Endpoint** | **Marker** | **AUC (95% CI)** | **Optimal cutoff** | **Sensitivity, %** | **Specificity, %** |
| --- | --- | --- | --- | --- | --- |
| Major complication | POD1 CALLY | 0.853 (0.783-0.915) | ≤0.0292 | 82.5 | 78.4 |
| Major complication | POD3 CALLY | 0.953 (0.920-0.979) | ≤0.0111 | 85.0 | 91.9 |
| Major complication | POD7 CALLY | 0.988 (0.974-0.997) | ≤0.0236 | 97.5 | 92.6 |
| Major complication | Discharge CALLY | 0.960 (0.928-0.985) | ≤0.0367 | 87.5 | 91.2 |
| Adapted textbook outcome failure | POD1 CALLY | 0.837 (0.765-0.897) | ≤0.0292 | 75.5 | 79.9 |
| Adapted textbook outcome failure | POD3 CALLY | 0.933 (0.888-0.966) | ≤0.0163 | 91.8 | 79.9 |
| Adapted textbook outcome failure | POD7 CALLY | 0.967 (0.943-0.986) | ≤0.0236 | 85.7 | 94.2 |
| Adapted textbook outcome failure | Discharge CALLY | 0.948 (0.915-0.975) | ≤0.0430 | 83.7 | 91.4 |

**Note:** Recovery ratios were calculated as postoperative CALLY divided by preoperative CALLY and are presented as median (interquartile range). Longitudinal interaction tests were obtained from generalized estimating equation models fitted to natural-log-transformed recovery ratios with four postoperative time levels and an exchangeable working correlation structure; individual time-point summaries are descriptive. This complete-cohort sensitivity analysis included all 188 patients, including 10 patients who underwent laparoscopic gastrectomy. For ROC analyses, lower postoperative CALLY values indicated the adverse outcome; optimal cutoffs were selected using the Youden index, and AUC confidence intervals were estimated by bootstrap resampling. ROC results are exploratory discrimination analyses and should not be interpreted as prospective diagnostic validation. AUC, area under the receiver operating characteristic curve; CI, confidence interval; POD, postoperative day; CALLY, C-reactive protein-albumin-lymphocyte index.
