# Supplementary material for: Perioperative CALLY dynamics after curative gastrectomy for gastric adenocarcinoma: associations with nodal tumor burden, textbook surgical outcome, and disease-free survival
Source: Front Oncol. 2026 Sep 3;16:1915467. doi: 10.3389/fonc.2026.1915467 (PMC13581691; doi:10.3389/fonc.2026.1915467)
Supplement: Supplementary file 1 [file SupplementaryFile1.zip › Supplementary Table S7.docx]

**Supplementary Table S7. Sensitivity analysis using the original textbook surgical outcome definition with length of stay ≤11 days**

**Panel A. Textbook surgical outcome achievement according to length-of-stay definition**

| **Definition** | **Complete cohort (n=188)** | **Open-gastrectomy cohort (n=178)** |
| --- | --- | --- |
| Adapted definition with LOS <21 days | 139/188 (73.9) | 130/178 (73.0) |
| Original definition with LOS ≤11 days | 122/188 (64.9) | 115/178 (64.6) |

**Panel B. CALLY recovery ratios according to the original textbook surgical outcome definition in the open-gastrectomy cohort**

| **Time point** | **Achieved (n=115)** | **Not achieved (n=63)** |
| --- | --- | --- |
| POD1 | 0.0636 (0.0432–0.0947) | 0.0381 (0.0256–0.0596) |
| POD3 | 0.0388 (0.0268–0.0641) | 0.0158 (0.0087–0.0275) |
| POD7 | 0.1097 (0.0644–0.1698) | 0.0191 (0.0103–0.0539) |
| Discharge | 0.1494 (0.0857–0.2344) | 0.0489 (0.0145–0.1220) |

**Time-by-group interaction: Wald χ²(3)=279.0; p<0.001.**

**Panel C. Exploratory ROC discrimination for failure to achieve the original textbook surgical outcome in the open-gastrectomy cohort**

| **Marker** | **AUC (95% CI)** | **Optimal cutoff** | **Sensitivity, %** | **Specificity, %** |
| --- | --- | --- | --- | --- |
| POD1 CALLY | 0.802 (0.738–0.862) | ≤0.0275 | 63.5 | 85.2 |
| POD3 CALLY | 0.876 (0.815–0.922) | ≤0.0153 | 74.6 | 85.2 |
| POD7 CALLY | 0.927 (0.892–0.965) | ≤0.0458 | 90.5 | 80.0 |
| Discharge CALLY | 0.814 (0.740–0.878) | ≤0.0430 | 63.5 | 89.6 |

**Note:** The adapted textbook surgical outcome used LOS <21 days, whereas the original definition used LOS ≤11 days; all other composite components were unchanged. Panels B and C were restricted to patients undergoing open gastrectomy. Recovery ratios were calculated as postoperative CALLY divided by preoperative CALLY and are presented as median (interquartile range). The longitudinal model used natural-log-transformed recovery ratios; primary inference was based on the time-by-group interaction, and individual time-point summaries are descriptive. ROC analyses were exploratory; optimal cutoffs were identified using the Youden index, and AUC confidence intervals were estimated using bootstrap resampling. The original definition was retained only as a sensitivity analysis and was not used for primary inference. CALLY, C-reactive protein–albumin–lymphocyte index; LOS, length of stay; ROC, receiver operating characteristic; AUC, area under the receiver operating characteristic curve; CI, confidence interval; POD, postoperative day.
