# Supplementary material for: Perioperative CALLY dynamics after curative gastrectomy for gastric adenocarcinoma: associations with nodal tumor burden, textbook surgical outcome, and disease-free survival
Source: Front Oncol. 2026 Sep 3;16:1915467. doi: 10.3389/fonc.2026.1915467 (PMC13581691; doi:10.3389/fonc.2026.1915467)
Supplement: Supplementary file 1 [file SupplementaryFile1.zip › Supplementary Table S8.docx]

**Supplementary Table S8. Survival sensitivity analyses**

**Panel A. Complete curative-intent cohort**

| **Outcome** | **Overall (n=188)** | **Low CALLY (n=94)** | **High CALLY (n=94)** | **p value** |
| --- | --- | --- | --- | --- |
| Documented recurrence | 44 (23.4) | 27 (28.7) | 17 (18.1) | – |
| DFS event | 55 (29.3) | 35 (37.2) | 20 (21.3) | – |
| Death | 24 (12.8) | 15 (16.0) | 9 (9.6) | – |
| 1-year DFS | 85.9% | 78.8% | 93.1% |  |
| 2-year DFS | 68.7% | 61.0% | 76.4% |  |
| 3-year DFS | 62.9% | 57.6% | 68.1% |  |
| DFS log-rank | – | – | – | 0.012 |
| 1-year OS | 96.7% | 94.6% | 98.9% |  |
| 2-year OS | 91.3% | 88.2% | 94.5% |  |
| 3-year OS | 80.9% | 80.8% | 81.6% |  |
| OS log-rank | – | – | – | 0.134 |

**Panel B. Secondary sensitivity cohort with observed postoperative follow-up ≥12 months**

| **Outcome** | **Overall (n=159)** | **Low CALLY (n=76)** | **High CALLY (n=83)** | **p value** |
| --- | --- | --- | --- | --- |
| Documented recurrence | 40 (25.2) | 24 (31.6) | 16 (19.3) | – |
| DFS event | 46 (28.9) | 27 (35.5) | 19 (22.9) | – |
| Death | 18 (11.3) | 10 (13.2) | 8 (9.6) | – |
| 1-year DFS | 89.9% | 85.5% | 94.0% |  |
| 2-year DFS | 71.9% | 66.2% | 77.2% |  |
| 3-year DFS | 65.9% | 62.5% | 68.8% |  |
| DFS log-rank | – | – | – | 0.087 |
| 1-year OS | 100.0% | 100.0% | 100.0% |  |
| 2-year OS | 94.4% | 93.3% | 95.6% |  |
| 3-year OS | 83.6% | 85.5% | 82.5% |  |
| OS log-rank | – | – | – | 0.439 |

**Panel C. Disease-free survival Cox regression sensitivity estimates**

| **Cohort** | **CALLY variable** | **Hazard ratio (95% CI)** | **p value** |
| --- | --- | --- | --- |
| Complete cohort (n=188) | Low versus high CALLY, univariable | 1.99 (1.15–3.45) | 0.014 |
| Complete cohort (n=188) | Continuous CALLY, univariable | 0.47 (0.26–0.85) | 0.012 |
| Complete cohort (n=188) | Continuous CALLY, multivariable | 0.53 (0.29–0.96) | 0.037 |
| Observed follow-up ≥12 months (n=159) | Low versus high CALLY, univariable | 1.66 (0.92–2.99) | 0.091 |
| Observed follow-up ≥12 months (n=159) | Continuous CALLY, univariable | 0.46 (0.24–0.88) | 0.019 |
| Observed follow-up ≥12 months (n=159) | Continuous CALLY, multivariable | 0.51 (0.27–0.99) | 0.045 |

**Note:** Low and high CALLY groups were defined using the full-cohort median threshold of 0.572. DFS was measured from curative gastrectomy to documented recurrence, death from any cause, or last follow-up; OS was measured from curative gastrectomy to death from any cause or last follow-up. The multivariable DFS models included continuous preoperative CALLY, stage III disease, lymphovascular invasion, perineural invasion, and major postoperative complications; dichotomized and continuous CALLY were not entered simultaneously. The cohort restricted to observed postoperative follow-up ≥12 months was included only as a secondary sensitivity analysis because eligibility depends on the observed follow-up duration and may introduce survivorship selection. The 1-year OS estimate of 100% in Panel B reflects this conditioning and should not be interpreted as a population estimate. Primary survival inference was based on the calendar-defined follow-up-mature cohort reported in the main manuscript. CALLY, C-reactive protein–albumin–lymphocyte index; DFS, disease-free survival; OS, overall survival; HR, hazard ratio; CI, confidence interval.
