# Supplementary material for: Perioperative CALLY dynamics after curative gastrectomy for gastric adenocarcinoma: associations with nodal tumor burden, textbook surgical outcome, and disease-free survival
Source: Front Oncol. 2026 Sep 3;16:1915467. doi: 10.3389/fonc.2026.1915467 (PMC13581691; doi:10.3389/fonc.2026.1915467)
Supplement: Supplementary file 1 [file SupplementaryFile1.zip › Supplementary Table S9.docx]

**Supplementary Table S9. Paired comparison of CALLY with its individual components for selected clinical endpoints**

**Panel A. Preoperative markers**

| **Endpoint / time point** | **CALLY AUC** | **CRP AUC; p vs CALLY** | **Albumin AUC; p vs CALLY** | **Lymphocyte AUC; p vs CALLY** |
| --- | --- | --- | --- | --- |
| pN-positive disease, 127/188 events | 0.613 | 0.604; p = 0.693 | 0.584; p = 0.567 | 0.528; p = 0.022 |
| High LNR, 93/188 events | 0.631 | 0.609; p = 0.306 | 0.587; p = 0.420 | 0.560; p = 0.048 |
| High LODDS, 94/188 events | 0.634 | 0.615; p = 0.369 | 0.593; p = 0.446 | 0.554; p = 0.017 |
| DFS event, 52/165 events | 0.631 | 0.610; p = 0.404 | 0.521; p = 0.076 | 0.614; p = 0.665 |

**Panel B. Postoperative markers for major complications in the open-gastrectomy cohort (39/178 events)**

| **Endpoint / time point** | **CALLY AUC** | **CRP AUC; p vs CALLY** | **Albumin AUC; p vs CALLY** | **Lymphocyte AUC; p vs CALLY** |
| --- | --- | --- | --- | --- |
| POD1 | 0.849 | 0.955; p < 0.001 | 0.743; p = 0.004 | 0.722; p < 0.001 |
| POD3 | 0.951 | 0.976; p = 0.034 | 0.815; p < 0.001 | 0.866; p < 0.001 |
| POD7 | 0.987 | 0.993; p = 0.185 | 0.870; p < 0.001 | 0.927; p < 0.001 |
| Discharge | 0.958 | 0.919; p < 0.001 | 0.890; p < 0.001 | 0.930; p = 0.028 |

**Panel C. Postoperative markers for adapted textbook surgical outcome failure in the open-gastrectomy cohort (48/178 events)**

| **Endpoint / time point** | **CALLY AUC** | **CRP AUC; p vs CALLY** | **Albumin AUC; p vs CALLY** | **Lymphocyte AUC; p vs CALLY** |
| --- | --- | --- | --- | --- |
| POD1 | 0.832 | 0.902; p = 0.038 | 0.694; p < 0.001 | 0.727; p < 0.001 |
| POD3 | 0.930 | 0.934; p = 0.811 | 0.760; p < 0.001 | 0.858; p < 0.001 |
| POD7 | 0.967 | 0.962; p = 0.515 | 0.813; p < 0.001 | 0.919; p < 0.001 |
| Discharge | 0.945 | 0.888; p < 0.001 | 0.834; p < 0.001 | 0.925; p = 0.138 |

**Note:** Marker directions were oriented so that higher scores indicated higher-risk status. CRP values were used directly, whereas inverse CALLY, albumin, and lymphocyte values were used. Reported p values compare each individual component with CALLY using paired stratified bootstrap analysis with 5,000 resamples. No adjustment for multiple comparisons was applied; these analyses were exploratory. High LNR and high LODDS were defined as values above their exact full-cohort medians, displayed as 0.0526 and −2.605, respectively. CALLY, C-reactive protein–albumin–lymphocyte index; CRP, C-reactive protein; AUC, area under the receiver operating characteristic curve; LNR, lymph node ratio; LODDS, log odds of positive lymph nodes; DFS, disease-free survival; POD, postoperative day.
